# The Mediating Role of Coping Styles on Impulsivity, Behavioral Inhibition/Approach System, and Internet Addiction in Adolescents From a Gender Perspective

**DOI:** 10.3389/fpsyg.2019.02402

**Published:** 2019-10-24

**Authors:** Qi Li, Weine Dai, Yang Zhong, Lingxiao Wang, Bibing Dai, Xun Liu

**Affiliations:** ^1^Chinese Academy of Sciences, Key Laboratory of Behavioral Science, Institute of Psychology, Beijing, China; ^2^Department of Psychology, University of Chinese Academy of Sciences, Beijing, China; ^3^Center of Functionally Integrative Neuroscience and Positron Emission Tomography Center, Aarhus University, Aarhus, Denmark; ^4^Sino-Danish College, University of Chinese Academy of Sciences, Beijing, China; ^5^Sino-Danish Center for Education and Research, Beijing, China; ^6^Institute of Psychology, Tianjin Medical University, Tianjin, China

**Keywords:** adolescents, impulsivity, behavioral inhibition/approach system, coping styles, Internet addiction, gender differences

## Abstract

Previous findings have shown that impulsivity and Behavioral Inhibition/Approach System (BIS/BAS) have substantial effects on adolescents’ Internet addiction, but the mechanisms underlying these associations and gender differences in these effects have received little attention. We examined the mediating effects of coping styles from impulsivity, and BIS/BAS to Internet addiction as well as gender differences in these associations. A total of 416 Chinese adolescents were examined using a cross-sectional survey involving Young’s Diagnostic Questionnaire for Internet Addiction, Barratt Impulsiveness Scale, BIS/BAS scales, and Coping Style Scale for Middle School Students. The data were analyzed using the independent sample *t*-test, chi-square test, Pearson correlation, and structure equation modeling. The results from the multiple-group (by adolescent gender) structural model analysis revealed that both impulsivity (*p* < 0.001) and BIS (*p* = 0.001) directly predicted positive Internet addiction in girls, while both impulsivity (*p* = 0.011) and BAS (*p* = 0.048) directly predicted positive Internet addiction in boys. Furthermore, emotion-focused coping mediated the relationship between impulsivity and Internet addiction (β = 0.080, 95% CI: 0.023–0.168) and the relationship between BIS and Internet addiction (β = 0.064, 95% CI: 0.013–0.153) in girls, while in boys, problem-focused coping and emotion-focused coping mediated the association between impulsivity and Internet addiction (β = 0.118, 95% CI: 0.031–0.251; β = 0.065, 95% CI: 0.010–0.160, respectively) and problem-focused coping mediated the association between BAS and Internet addiction [β = −0.058, 95% CI: (−0.142)–(−0.003)]. These findings extend our insight into the mechanisms underlying the associations among impulsivity, BIS/BAS, and Internet addiction in adolescents and suggest that gender-sensitive training approaches to decrease adolescents’ Internet addiction are indispensable. These interventions should focus on the different gender predictors of adolescent Internet addiction and on the development of specific coping styles for boys and girls respectively.

## Introduction

With the speedy development of Internet technology in recent years, people increasingly use the Internet, especially adolescents. Although the growth in Internet use facilitates adolescent life in many ways, excessive Internet use can lead to Internet addiction (Choi et al., 2009; Ko et al., 2012). Internet addiction was defined as a subset of behavioral addictions that possess the core components of addiction, such as salience, tolerance, and withdrawal (Griffiths, 2000), while Shapira et al. (2000) described Internet addiction as an impulse control disorder. In 2008, Shaw and Black (2008) further refined the conception of “Internet addiction” as “excessive or poorly controlled preoccupations, urges or behaviors regarding computer use and Internet access that lead to impairment or distress.” In consideration of the variety of terminologies, the lack of consistency about the conceptualization and the diagnosis of Internet addiction, Sim et al. (2012) have suggested that the most reliable and valid criterion for conceptualizing Internet addiction is to adapt the Diagnostic and Statistical Manual of Mental Disorders, 4th Edition (DSM- IV) criteria for pathological gambling. Consistent with this notion, Young defined Internet addiction as an individual’s inability to control the impulse to use their Internet use, which eventually leads to psychological, social, educational, and/or occupational problems (Young, 1998). Furthermore, she confirmed 8 symptoms of Internet addiction according to the criteria for pathological gambling in the DSM-IV: tolerance, preoccupation, withdrawal symptoms, unsuccessful attempts to decrease use, continued excessive use, compromise or loss of a significant relationship and social activities, lying about online activity, and use of the Internet to self-medicate. Meanwhile, she developed a brief eight-item Diagnostic Questionnaire for Internet Addiction (YDQ) to assess Internet addiction (Young, 1998). Although Internet addiction has not yet been classified as a disorder, neither in the DSM-V nor in the eleventh edition of the International Classification of Diseases (ICD-11), most of the items in YDQ directly corresponded to the nine diagnostic criteria for Internet Gaming Disorder in the DSM-V (American Psychiatric Association [APA], 2013). Furthermore, “chemical” addiction showed no difference with “behavioral” addiction according to DSM-V addiction criteria, and DSM-V paid more attention to personal experiences rather than drug types (American Psychiatric Association [APA], 2013). Subsequently, Internet Gaming Disorder was included as “Disorders due to addictive behaviors” rather than as an “Impulse Control Disorder” in the ICD-11 (World Health Organization [WHO], 2018).

Adolescence is a critical stage in life cycle, and can be defined as a transitional period from childhood to adulthood during which individuals experience major biological, cognitive, and socioaffective changes (Dumont and Provost, 1999). Consequently, adolescents have to cope with the most stressful life-events and the challenges caused by these changes. They face specific developmental tasks including identity construction, personal autonomy, and the redefinition of relationships with adults and peers (Borca et al., 2015). Internet addiction is highly prevalent and causes more harmful consequences among adolescents than among adults in many countries due to their active psycho-social and personality development (Morrison and Gore, 2010; Spada, 2014; Stavropoulos et al., 2017). The data from a nationally representative sample of Chinese adolescents indicated that the percentage of Internet addicts in the total sample (Internet users and non-Internet users) was 6.3% (1,523/24,013), while among Internet users, it was 11.7% (1,523/12,993) (Li et al., 2014). In addition, the prevalence of Internet addiction is much higher in Asian countries (e.g., China) than in Western countries (Li et al., 2018). Moreover, Internet addiction is significantly correlated with numerous negative consequences in adolescents, such as psychiatric disorders (e.g., depression, anxiety and obsessive-compulsive specifications), physical problems, and poor academic performance (Ko et al., 2009; Salmela-Aro et al., 2017; Przepiorka et al., 2019). More importantly, addiction and its’ negative influences in adolescents could continue into adulthood (Englund et al., 2008; Stavropoulos et al., 2017). Thus, it is very important to study Internet addiction among adolescents. To promote the prevention of and early intervention for Internet addiction, it is imperative to identify risk factors and underlying mechanisms for Internet addiction in adolescents, especially among those in Asian countries.

The dual-system neurobiological model tries to explain high risk-taking behaviors in adolescents, such as substance abuse, pathological gambling, Internet gaming disorders, and so on (Casey et al., 2008). It proposes that the differential development of the reward-seeking and impulse control system in adolescents, which show heightened reward-seeking and deficient impulse control relative to children and adults, might be one of the most important factors contributing to the high incidence of adolescents’ risky behaviors (Steinberg, 2010). This model goes against traditional explanations, which states that risky behaviors in adolescents are mainly due to a lag in the development of the prefrontal control system and advocates to combine the development of the subcortical reward-seeking system with that of the prefrontal control system (Rubia et al., 2000, 2006; Tamm et al., 2002; Yurgelun-Todd, 2007). In line with the dual-system model, many neurobiological models indicate that the reward system and impulse control system have equal importance in terms of accounting for adolescent’s risky behaviors, including substance abuse and problematic gambling (Casey et al., 2008; Steinberg, 2008; Somerville and Casey, 2010). However, few studies have simultaneously explored the characteristics of reward processing and impulse control in the context of Internet addition among adolescents to test and extend the dual-system model for this condition.

### Impulsivity, BIS/BAS and Internet Addiction

Impulsivity is defined as a predisposition that leads to the tendency to behave prematurely and without foresight in ways that are undesirably dangerous or unsuitable to the situation (Robbins et al., 2012). Impulsivity is often associated with the inhibitory control systems due to an immature frontal lobe that causes adolescents to be at particularly high risk for Internet addiction (Crews and Boettiger, 2009; Brand et al., 2014). Previous studies have revealed that the high level of Internet addiction among adults is positively associated with impulsivity (Meerkerk et al., 2010; Zhang et al., 2015), and adolescents with Internet addiction exhibit increased impulsivity and reduced inhibitory control capacity compared with controls (Cao et al., 2007; Choi J. S. et al., 2014; Choi S.-W. et al., 2014; Bargeron and Hormes, 2017). Many researchers have emphasized the key role of impulsivity in Internet addiction and have argued that impulsivity is an important risk factor for developing Internet addiction and a marker of susceptibility to Internet addiction (Lee et al., 2012; Wu et al., 2013; Li et al., 2016).

Gray’s neuropsychological reinforcement sensitivity theory states that behavior originates from activity in at least two basic dimensions of motivation, which are independent and based on biological systems (Gray, 1994; Bijttebier et al., 2009). These dimensions reveal the function of two brain systems that govern approach and avoidance behaviors in response to different types of stimuli. The behavioral approach system (BAS) is responsible for mediating reactions to all conditioned and unconditioned appetitive stimuli and is associated with the enhancement of reward or the termination of punishment. The BAS is associated with reward seeking and high levels of BAS activation indicate higher sensitivity to reward dependence and novelty processing (Li et al., 2019). The behavioral inhibition system (BIS) has been postulated to be sensitive to stimuli of punishment or the termination of a reward. The BIS is associated with the avoidance of potentially negative or harmful consequences and high levels of BIS activation imply a proneness to loss avoidance and a tendency to display a blunted response to reward (Li et al., 2019). Although Gray’s neuropsychological reinforcement sensitivity theory provided an important view for understanding and explaining addiction, previous results about the associations among BIS/BAS and Internet addiction were inconsistent both among adults and among adolescents. The BIS is neither directly nor indirectly associated with Internet addiction, whereas the BAS is associated with Internet addiction only through depression and social anxiety in adults (Fayazi and Hasani, 2017), while another study found that neither the BAS and nor BIS were related to Internet addiction in adults (Meerkerk et al., 2010). In terms of adolescents, high BAS activation rather than BIS activation could predict the occurrence of Internet addiction (Yen et al., 2012), while another study indicated that the BIS activation not the BAS activation was a significant predictor of Internet addiction (Park et al., 2013). Further, two previous studies reported that both high BAS and high BIS activation were associated with Internet addiction (Giles and Price, 2008; Nam et al., 2018). Although these results from adolescents could indicate that Gray’s neuropsychological reinforcement sensitivity theory can help to understand and explain Internet addiction, the inconsistent results require more research to explore the associations between BIS/BAS and Internet addiction. In sum, few studies have explored the roles that impulsivity and BIS/BAS play in Internet addition among adolescents, especially on the basis of the dual-system neurobiological model of addiction. Furthermore, although most previous studies have shown that impulsivity and BIS/BAS contribute to Internet addiction among adolescents, little is known about the mediating and moderating mechanisms underlying these associations.

### The Mediating Role of Coping Style

Coping style refers to people’s behavioral and cognitive attempts to manage specific external and/or internal demands under stress (Skinner et al., 2003). In general, coping styles can be divided into problem-focused and emotion-focused coping. Problem-focused coping refers to strategies that are directed to address the problems that cause emotional distress (e.g., problem solving, seeking help, and cognitive restructuring), whereas emotion-focused coping refers specifically to strategies that palliate negative emotions (e.g., wishful thinking, denial, and withdrawal behavior) (Compas et al., 2001). Because both coping styles show contextual and process-oriented features, they can change over time and under different circumstances (Schoenmakers et al., 2015). Furthermore, how coping styles are used depends on how an individual interprets the stressor, and different coping styles could cause different results. Taken together, adolescents with high problem-focused coping tend to find appropriate methods to address their difficult circumstances, accompanied by better adjustment (Jackson et al., 2017), while adolescents with high emotion-focused coping tend to avoid their own problems passively, accompanied by maladjustment (Carlo et al., 2012). In previous studies, researchers noted that the two types of coping should be viewed as different constructs of coping instead of opposite poles (Patterson and McCubbin, 1987; Compas et al., 2001). Although there is no direct empirical evidence supporting that coping mediates the relationships among impulsivity, BIS/BAS and Internet addiction in adolescents, some indirect evidence has implied that coping styles play a mediating role in these associations. On one hand, previous studies have found that adolescents with high impulsivity are more likely to use emotion-focused coping but less likely to engage in problem-focused coping (Connor-Smith and Flachsbart, 2007; Lee-Winn et al., 2016). The BAS not the BIS was associated with problem-focused coping, and problem-focused coping mediated the relationship between the BAS and adolescent delinquent behavior, while another study found that emotion-focused coping mediated the relationship between the BIS and adolescent problematic alcohol use (Hasking, 2007; Willem et al., 2012). On the other hand, both high emotion-focused coping and low problem-focused coping were associated with adolescent Internet addiction, while another study found that only emotion-focused coping, not problem-focused coping, increased the risk of Internet addiction in adolescence (Tang et al., 2014; Zhou et al., 2017). Thus, impulsivity and BIS/BAS may be associated with coping styles, which in turn could be associated with Internet addiction. However, to the best of our knowledge, no empirical study has directly explored whether coping styles mediate the relationships among impulsivity, BIS/BAS and adolescent Internet addiction.

### The Moderating Role of Gender

Gender is a factor that potentially moderates the links among impulsivity, BIS/BAS, coping styles and Internet addiction. First, previous studies have shown that there are significant gender differences in some of the above variables. For example, men have a relatively higher tendency toward Internet addiction than women in a meta-analysis involving 34 global jurisdictions, suggesting that gender-related differences in Internet availability and social norms could account for the gender differences in Internet addiction (Su et al., 2019). Specifically, greater gender gap in Internet penetration are associated with larger effect sizes of gender differences in Internet addiction (*B* = 0.223, 95% CI: 0.086–0.360). Furthermore, the more the social norms preferentially approve men to involve in potentially addictive behaviors such as smoking and alcohol consumption, the more men exhibit higher tendencies of Internet addiction than women (Su et al., 2019). The prevalence of Internet addiction has been found to be higher in boys than in girls (Ha and Hwang, 2014). Boys have higher impulsivity than girls (Munno et al., 2016). Two previous studies found that women have higher BIS activation and higher activation of specific aspects of the BAS (reward responsiveness) than men (Jorm et al., 1998; Nam et al., 2018). Women were more likely to use emotion-focused coping and less likely to use problem-focused coping than men (Matud, 2004). More importantly, it has been suggested that there are gender differences in the pathway associations between these variables. Compared with girls, Internet addiction has a strong association with impulsivity in boys (Nam et al., 2018). A meta-analysis of 46,025 adolescents found that gender is an important factor that could moderate the relationships between coping styles and Internet addiction in Chinese adolescents (Lei et al., 2018). Furthermore, girls with emotional difficulties were more easily affected by Internet addiction than boys with similar problems (Ha and Hwang, 2014). In addition, according to the social gender role theory, men are socialized as independent and self-reliant while women are socialized as warm, supportive, compassionate, sensitive to the feelings of others, and emotionally expressive (Reevy and Maslach, 2001), which further provides potential evidence for gender as a moderator among impulsivity, BIS/BAS, coping styles and Internet addiction.

### The Present Study

**FIGURE 1:**
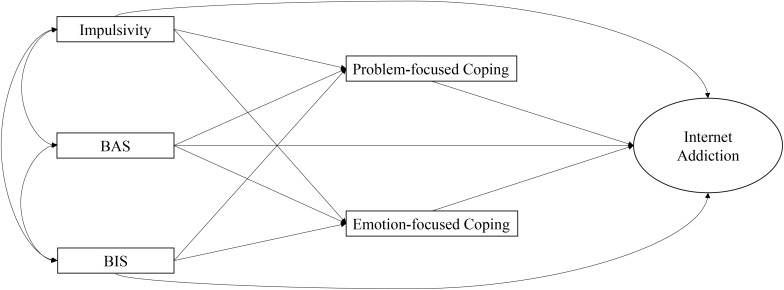
The supposed model.

The purpose of the present study was to examine how impulsivity and BIS/BAS influence Internet addiction in adolescents. Specifically, this study explored the mediating effects of coping styles on impulsivity, BIS/BAS and Internet addiction as well as the gender differences among these associations. To our knowledge, this is the first comprehensive empirical study incorporating impulsivity, BIS/BAS, coping styles and gender factors and their roles in Internet addiction. On the basis of the dual-system neurobiological model and gender social roles theory (Reevy and Maslach, 2001; Casey et al., 2008), the proposed model is presented in Figure 1. It is plausible to hypothesize that coping styles act as mediator among impulsivity, BIS/BAS and Internet addiction, and gender serves as a moderator among these associations in adolescents. More specifically, we want to examine whether the dual-system neurobiological model includes high impulsivity and high BIS in Internet addiction among girls, while the dual-system neurobiological model combines high impulsivity and high BAS in boys. Furthermore, we suggest that emotion-focused coping will play an important role in girls, while problem-focused coping will be central in boys. Therefore, our hypotheses are as follows: (1) boys will report greater Internet addiction than girls; (2) impulsivity and the BIS will positively predict Internet addiction in girls; (3) impulsivity and the BAS will positively predict Internet addiction in boys; (4) emotion-focused coping will serve as a mediator between impulsivity and the BIS with Internet addiction in girls; and (5) problem-focused coping will serve as a mediator between impulsivity and the BAS with Internet addiction in boys.

## Materials and Methods

### Participants and Procedure

According to the rule of thumb approach in the structural equation modeling, 10:1 is the commonly suggested ratio of sample size to free parameters, which is often used for minimum recommendations to determine a sample size for a structural equation modeling test (Bentler and Chou, 1987; Kline, 2016). Because 42 was the largest number of free parameters in our all structural equation models, the sample size was decided to be more than 420 in the present study. A total of 450 Chinese adolescents were recruited from four public schools in Beijing City, China. Among these adolescents, 416 participants (*M_*age*_* = 14.56 years, *SD* = 1.42 years, age range: 11–18 years) completed the questionnaires, for a response rate of 92.44%. Thirty-four cases were excluded prior to analysis due to unreturned forms. The sample included 212 girls and 204 boys; 139 (33.41%) of the students were in the 7th grade, 98 (23.56%) in the 8th grade, 102 (24.52%) in the 10th grade, and 77 (18.51%) in the 11th grade. More detailed demographic information was shown in Table 1. All participants were fluent in Mandarin.

**TABLE 1:** Descriptive statistics among the variables (*N* = 416).

**Variables**	**Girls (*N* = 212)**			**Boys (*N* = 204)**			***t*-test**
			
	**Range**	***M***	***SD***	**Range**	***M***	***SD***	
Age (years)	12–17	14.52	1.34	11–18	14.61	1.50	–0.67
Education level (years)	7–11	8.67	1.49	7–11	8.75	1.66	–0.49
Network age (years)	0–13	4.94	2.56	0–14	5.06	2.96	–0.44
Internet usage time (hours)	0–10	1.64	1.79	0–14	1.65	2.04	–0.07
Online game time (hours)	0–7	0.36	0.86	0–9	0.88	1.45	–4.36^∗∗^
Internet addiction	0–8	2.06	1.81	0–8	2.33	1.93	–1.49
Impulsivity	37–131	70.64	14.45	30–147	71.89	16.39	–0.83
BAS	29–52	43.67	5.29	29–52	42.51	5.18	2.24^∗^
BIS	11–28	20.82	3.25	14–28	20.03	2.85	2.63^∗∗^
Problem-focused coping	35–75	58.62	8.23	19–75	57.01	10.29	1.76
Emotion-focused coping	18–68	40.33	7.94	17–62	39.60	8.01	0.93

**^∗^p < 0.05; ^∗∗^p < 0.01.**

### Ethics Statement

The present study was approved by the Ethics Committee of the Institute of Psychology of the Chinese Academy of Sciences. School approval and parental consent were obtained prior to originating the study. All participants were informed that they could quit the study at any time without being penalized.

### Measures

#### Internet Usage

For each participant, Internet usage information was obtained using 4 questions: (1) “How many years have you used the Internet?” (2) “How many hours do you use the Internet every day?” (3) “What is your duration of online gaming every day?” and (4) “Do you often play online gaming (Yes or No)?”

#### Young’s Diagnostic Questionnaire for Internet Addiction (YDQ)

The YDQ was applied to assess Internet addiction. The YDQ was modified according to the DSM-IV criteria for pathological gambling and consists of 8 “yes” or “no” questions (e.g., “Do you feel the need to use the Internet with increasing amounts of time in order to achieve satisfaction?” and “Do you feel restless, moody, depressed, or irritable when attempting to cut down or stop Internet use?”) (Young, 1998). Total scores were calculated according to Young’s method, with possible scores for all 8 items ranging from 0 to 8. Higher scores reflect a higher level of Internet addiction. Because the YDQ is one of the most widely used questionnaires to evaluate Internet addiction, it has also good reliability and validity in Chinese adolescents (Cao et al., 2007; Li et al., 2014). In the present study, confirmatory factor analysis of the unidimensional model indicated that the model for Internet addiction showed a good fit with the data: χ^2^/df = 1.966, *p* < 0.05; CFI = 0.949, TLI = 0.928, RMSEA = 0.048 and SRMR = 0.041. In the current study, Cronbach’s alpha coefficient for Internet addiction was 0.66.

#### Barratt Impulsiveness Scale (BIS-11)

The BIS-11 is widely used to assess participants’ impulsive traits by rating their frequency of 30 items on a scale from 1 (never) to 4 (always) (Patton et al., 1995). The BIS-11 includes three impulsiveness subscales: cognitive key (e.g., “I get easily bored when solving thought problems”), motor key (e.g., “I say things without thinking”), and non-planning key (e.g., “I am more interested in the present than in the future”) (Choi J. S. et al., 2014; Martinez-Loredo et al., 2015). The overall impulsiveness score is determined by summing all items, with higher scores denoting greater impulsivity. In the present study, the Chinese version of the BIS-11 was used (Lu et al., 2012), and Cronbach’s alpha coefficient was 0.91.

#### Behavioral Inhibition System/Behavioral Approach System (BIS/BAS) Scales

A validated Chinese version of the BIS/BAS scales was used to assess the BIS and BAS (Li et al., 2015). The BIS/BAS scales are comprised of 20 items in addition to 4 filler items and include the Behavioral Approach System Scale (BAS, 13 items) and the Behavioral Inhibition System Scale (BIS, 7 items) (Carver and White, 1994). The former scale can be divided into three subscales: drive (BAS-drive, 4 items), reward responsiveness (BAS-reward, 5 items), and fun seeking (BAS-fun, 4 items). All items were assessed on a 4-point Likert scale from 1 (totally disagree) to 4 (totally agree). Sample items are “When I see an opportunity for something I like I get excited right away (BAS)” and “Criticism or scolding hurts me quite a bit (BIS).” In the present study, the Cronbach’s alpha coefficients of the BAS-drive, BAS-reward, and BAS-fun were 0.67, 0.67, and 0.62, respectively. Scores for all 13 BAS items were summed to yield a single BAS score. Only the total BAS score was used in the current study. The Cronbach’s alpha coefficients for the BAS and BIS in the current sample were 0.80 and 0.58, respectively.

#### Coping Style Scale for Middle School Students

Adolescent coping styles were assessed with the Coping Style Scale for Middle School Students, which has been adapted for the Chinese culture (Dumont and Provost, 1999; Zhou et al., 2017; Sun et al., 2019). This inventory was designed on the basis of Folkman’s interaction theory, the self-regulation theory and a prior coping styles questionnaire (Folkman et al., 1986). It is divided into two categories based on coping style, problem-focused coping and emotion-focused coping (Folkman et al., 1986), and includes 36 items rated on a 4-point Likert scale from 1 (never coping) to 4 (often coping). Problem-focused coping consists of three subscales, including problem solving (7 items, e.g., “I make a plan to solve problems and execute it step by step”), support seeking (7 items, e.g., “I strive to get advice from someone about what to do”), and reasonable explanation (5 items, e.g., “I try to change my perspective to explore the positive side of frustration”). Emotion-focused coping consists of four subscales, including tolerance (4 items, e.g., “My ability is limited, so the only things I can do about unpleasant things is tolerate them”), avoidance (4 items, e.g., “I admit that I can’t deal with a problem at hand, so I will give up trying”), venting emotions (4 items, e.g., “I express emotions to reduce my unhappiness”), and fantasy/denial (5 items, e.g., “I say to myself ‘this isn’t real’ when encountering difficulties”). The scale has high construct validity, discrimination validity, and reliability in Chinese adolescents (Chen et al., 2000). In the current study, Cronbach’s alpha coefficients for problem-focused coping and emotion-focused coping were 0.88 and 0.79, respectively.

### Procedure

The participants were given a packet of questionnaires that included instructions on how to respond to the questions and assurances of anonymity as well as questions regarding their basic demographic information, including gender, age, education grade, BIS/BAS, impulsivity, and coping styles. All scales were administered to participants in their classes. Students were tested individually in their classrooms. All the questionnaires were printed in the Chinese language and took approximately 30 min to finish. No personal identifying information was collected, and all the information collected was confidential.

### Data Analysis

Because the proportion of missing data was very low (<1%), mean substitution was adopted to deal with missing data. First, SPSS 20.0 was used to compute descriptive statistics and perform correlation analyses, Chi-square test and *t*-tests. Next, Amos 21.0 was used to test the hypothesized models. Structural equation modeling (SEM) was conducted to test the mediating role of coping styles in the relationships among impulsivity, BIS/BAS and Internet addiction. Furthermore, to assess gender differences, a multi-group (by adolescent gender) SEM was used.

In the present study, several goodness-of-fit indices were used to test the model-data fit. The first one was the Chi-square statistic and its associated *p*-value. If the *p*-value is not significant, it may indicate good model-data fit. However, the Chi-square statistic is sensitive to sample size (Bollen, 1989). Therefore we used the Chi-square to degrees of freedom ratio (χ^2^*/df*) to test model fit. A χ^2^*/df* ratio of less than 3 shows an admissible model fit. Other substitutive indices were also employed in the current study, including the comparative fit index (CFI) (Rigdon, 1996), the Tucker-Lewis Index (TLI) (Tucker and Lewis, 1973), the root mean square error of approximation (RMSEA) (Browne and Cudeck, 1993) and the standardized root mean square residual (SRMR) (Hooper et al., 2008). A CFI and TLI larger than 0.95 and a RMSEA and SRMR less than 0.08 show good model fit (Hooper et al., 2008). For the comparison of the nested models, differences in the χ^2^ *(△*χ^2^) and the degree of freedom (*△df*) were used to compare the models with the goodness of fit to determine the model that best fit the data (Satorra, 2000; Byrne and Stewart, 2006). Specifically, the standard of comparison between the two nested models is as follows: when the degrees of freedom increase without a significant increase in the corresponding Chi-square value (that is, *△*χ^2^*/△df* is not significant), the better model is the one with a larger degrees of freedom. Otherwise, the smaller degrees of freedom model is better. The predictive and explanatory powers of the model were measured using path coefficients and *R*^2^.

## Results

### Descriptive Statistics and *t*-Tests/χ^2^-Test

The ranges, means and standard deviations of the continuous variables are shown in Table 1 for girls and boys separately. There were no gender differences in age, education level, network age, Internet usage time, Internet addiction, impulsivity or coping, although boys showed a longer online gaming time. In the present study, 8.5% (*n* = 18) of girls frequently played online games, while 34.3% (*n* = 70) of boys frequently played online games. These results indicate that boys are more frequent online gamers than girls (χ*^2^* = 41.27, *p* < 0.001). Furthermore, BIS/BAS yielded significant gender differences. Compared with girls, boys had lower scores on the BIS and the BAS scales.

### Correlation Analyses

Table 2 presents the correlations among the variables in the current study, with girls above the diagonal and boys below the diagonal. For both girls and boys, Internet addiction was positively related to impulsivity, BIS and emotion-focused coping, while Internet addiction was only negatively related to problem-focused coping in boys but had no correlations in girls. For both girls and boys, impulsivity was significantly related to problem-focused coping and emotion-focused coping. There were significant correlations between BAS and both problem-focused coping and emotion-focused coping in boys, but these correlations were not present in girls. For both girls and boys, BIS was positively related to emotion-focused coping but was not significantly related to problem-focused coping. Furthermore, there were significant gender differences in the correlations based on a one-tailed *z*-test, with a stronger association between Internet addiction and BIS (*z* difference = 1.73) for girls than for boys (*p* < 0.05), a stronger association between Internet addiction and problem-focused coping (*z* difference = 2.80) for boys than for girls (*p* < 0.01), a stronger association between impulsivity and problem-focused coping (*z* difference = 3.08) for boys than for girls (*p* < 0.01), a stronger association between BAS and problem-focused coping (*z* difference = 1.91) for boys than for girls (*p* < 0.05), and a stronger association between BIS and emotion-focused coping (*z* difference = 1.73) for girls than for boys (*p* < 0.05). The following analyses of the hypothesized models were executed based on the correlation models of these variables.

**TABLE 2:** Associations among the variables for girls and boys.

**Variable**	**1**	**2**	**3**	**4**	**5**	**6**
Internet addiction	–	0.39^∗∗^	0.12	0.32^∗∗^	0	0.38^∗∗^
Impulsivity	0.36^∗∗^	–	0.23^∗∗^	0.22^∗∗^	–0.24^∗∗^	0.37^∗∗^
BAS	0.11	–0.09	–	0.18^∗∗^	0.12	0.10
4. BIS	0.16^∗^	0.09	0.24^∗∗^	–	0.07	0.32^∗∗^
Problem-focused coping	–0.27^∗∗^	–0.50^∗∗^	0.30^∗∗^	0.08	–	0.06
Emotion-focused coping	0.28^∗∗^	0.37^∗∗^	0.21^∗∗^	0.16^∗^	–0.05	–

**BAS, Behavioral Approach System; BIS, Behavioral Inhibition System; ^∗^p < 0.05; ^∗∗^p < 0.01.**

### Structural Equation Model Analyses

**FIGURE 2:**
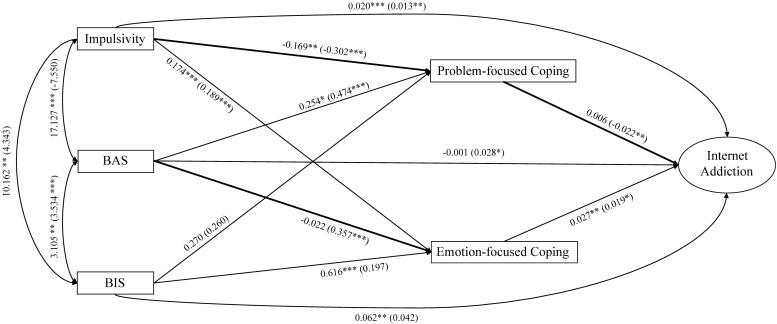
The relationships among impulsivity, BAS, BIS and Internet addiction mediated by coping. Bold lines indicate significant gender differences in these paths. The parameters for girls are displayed outside of the parentheses, while the parameters for boys are denoted within the parentheses. ^∗^*p* < 0.05, ^∗∗^*p* < 0.01, and ^∗∗∗^*p* < 0.001.

Before analyzing the structural equation model, five observed variables (impulsivity, BAS, BIS, problem-focused coping, and emotion-focused coping, representative of their total scores respectively) and one latent variable (Internet addiction) were used to make our model more simplified and efficient. Furthermore, the YDQ was divided into two parcels, where the sum of items 1, 3, 5, and 7 constituted the first parcel (parcel 1), and the sum of items 2, 4, 6, and 8 constituted the second parcel (parcel 2), to act as indicators of Internet addiction employing an item-to-construct balance approach (Little et al., 2002). Then, structural equation modeling with AMOS 21.0 was carried out to examine our hypothesized mediation model. The factor loadings of Internet addiction for parcel 1 and parcel 2 were 0.799 and 0.689, respectively. The results of the model showed a good fit with the data: χ*^2^/df* = 2.417, *p* < 0.05; CFI = 0.984, TLI = 0.935, RMSEA = 0.058 and SRMR = 0.024. To test whether gender moderated the path relationships among these variables, two nested models were estimated. Specifically, we examined whether the estimate of the model parameters (i.e., path coefficients) varied between girls and boys. The first model permitted the structure coefficient of the two models to be estimated freely according to gender, while the second model was administered for the structure path coefficient to be equal. The results showed that these two models were significantly different, △χ*^2^* (11, N = 416) = 25.424, *p* = 0.008, indicating that they differed according to gender. The structural model displaying unstandardized regression coefficients between variables is presented in Figure 2. In addition, we utilized critical ratios of differences (CRDs) as an index to examine the differences in structural path coefficients between genders. If the CRD was larger than 1.96, then the associations between these two variables would demonstrate a significant gender difference at *p* < 0.05. The results showed that the structure path from impulsivity to problem-focused coping revealed a significant gender difference (CRD = 2.48, *p* < 0.05). More specifically, the path coefficient for girls was β = −0.30, *p* < 0.001, while the path coefficient for boys was β = −0.48, *p* < 0.001. Thus, compared with girls, impulsivity had a far greater negative influence on problem-focused coping among boys. The structure path from problem-focused coping to Internet addiction also revealed a significant gender difference (CRD = 2.51, *p* < 0.05). More specifically, the path coefficient for girls was β = 0.06, *p* > 0.05, while the path coefficient for boys was β = −0.25, *p* < 0.01. Therefore, problem-focused coping had a far greater negative prediction to Internet addiction among boys than among girls. Furthermore, the structure path for the path from BAS to emotion-focused coping revealed a significant gender difference (CRD = −2.73, *p* < 0.05). More specifically, the path coefficient for girls was β = −0.02, *p* > 0.05, while the path coefficient for boys was β = 0.23, *p* < 0.001. This result suggests that BAS had a far greater positive impact on emotion-focused coping among boys than among girls. The unstandardized regression coefficients from the multiple-group structural model analysis and the CRDs between girls and boys are presented in Table 3. In total, the model explained 32.8% of the variance in Internet addiction among girls and 30.7% of the variance in Internet addiction among boys.

**TABLE 3:** Unstandardized coefficients from the multiple-group analysis.

**Structural model**	**Girls estimate (*S.E.*)**	**Boys estimate (*S.E.*)**	**CRD**
Impulsivity to Internet addiction	0.020 *(0.005*)^∗∗∗^	0.013 (*0.005*)^∗∗^	0.933
BAS to Internet addiction	−0.001 (*0.012*)	0.028 (*0.014*)^∗^	–1.549
BIS to Internet addiction	0.062 (*0.021*)^∗∗^	0.042 (*0.024*)	0.627
Impulsivity to problem-focused coping	−0.169 (*0.039*)^∗∗∗^	−0.302 (*0.037*)^∗∗∗^	2.484^∗^
BAS to problem-focused coping	0.254 (*0.105*)^∗^	0.474 (*0.120*)^∗∗∗^	–1.383
BIS to problem-focused coping	0.270 (*0.172*)	0.260 (*0.217*)	0.038
Impulsivity to emotion-focused coping	0.174 (*0.035*)^∗∗∗^	0.189 (*0.031*)^∗∗∗^	–0.326
BAS to emotion-focused coping	−0.022 (*0.096*)	0.357 (*0.101*)^∗∗∗^	−2.728^∗^
BIS to emotion-focused coping	0.616 (*0.156*)^∗∗∗^	0.197 (*0.183*)	1.745
Problem-focused coping to Internet addiction	0.006 (*0.008*)	−0.022 (*0.008*)^∗∗^	2.509^∗^
Emotion-focused coping to Internet addiction	0.027 (*0.009*)^∗∗^	0.019 (*0.009*)^∗^	0.644

**The numbers in italics in the parentheses represent the standard errors; S.E., standard error; CRD, critical ratio difference.**

When the final model was chosen, bias-corrected bootstrapping, a non-parametric resampling procedure, was utilized to further test the significance of the mediators. Bootstrapping has considerably greater statistical power to test indirect effects than traditional mediation analyses (MacKinnon et al., 2004). When the 95% confidence intervals (CIs) do not include zero, the indirect effect is statistically significant. In the present study, 5000 bootstrapping samples were generated to derive CIs. The results of the bootstrap analyses indicated that the specific indirect effect of impulsivity on Internet addiction through emotion-focused coping was significant, and the total indirect effects of BIS on Internet addiction and the specific indirect effect of BIS on Internet addiction through emotion-focused coping were also significant in girls. The total indirect effects of impulsivity on Internet addiction and the specific indirect effects of impulsivity on Internet addiction through problem-focused coping or emotion-focused coping were significant, and the specific indirect effect of BAS on Internet addiction through problem-focused coping was also significant in boys (see Table 4).

**TABLE 4:** The bootstrapping results of the indirect effects in the final model.

**Model paths**	**Standard indirect effects**	**95% CI**	
		
		**Lower**	**Upper**
**GIRLS**			
Impulsivity→ Internet addiction^a^	0.063	–0.028	0.172
Impulsivity → PFC → IA	–0.017	–0.072	0.033
Impulsivity → EFC→ IA	0.080^∗^	0.023	0.168
BAS → Internet addiction^a^	0.005	–0.060	0.057
BAS → PFC → IA	0.009	–0.019	0.043
BAS → EFC → IA	–0.004	–0.054	0.032
BIS → Internet addiction^a^	0.070^∗^	0.015	0.162
BIS → PFC → IA	0.006	–0.010	0.043
BIS → EFC → IA	0.064^∗^	0.013	0.153
**BOYS**			
Impulsivity→ Internet addiction^a^	0.183^∗∗^	0.093	0.326
Impulsivity → PFC → IA	0.118^∗^	0.031	0.251
Impulsivity → EFC → IA	0.065^∗^	0.010	0.160
BAS → Internet addiction^a^	–0.019	–0.117	0.067
BAS → PFC → IA	−0.058^∗^	–0.142	–0.003
BAS→ EFC → IA	0.039	–0.002	0.101
BIS → Internet addiction^a^	–0.006	–0.063	0.041
BIS→ PFC → IA	–0.018	–0.071	0.014
BIS→ EFC → IA	0.012	–0.007	0.068

**^*a*^The indirect effect represents the effect through all possible mediators (i.e., problem-focused coping, emotion-focused coping). CI, confidence interval; PFC, problem-focused coping; EFC, emotion-focused coping; IA, Internet addiction. ^∗^p < 0.05, ^∗∗^p < 0.01.**

## Discussion

### Gender Differences in Internet Addiction

Contrary to our hypothesis, we did not find gender differences in Internet addiction, which was in line with the results of McNicol and Thorsteinsson (2017). According to the Internet Availability hypothesis (Mann, 2005), availability is an important determinant of addictive behavior. Men have higher levels of Internet addiction than women, which may be associated in part with gender-related differences in Internet availability (Su et al., 2019). A recent meta-analysis involving 34 global jurisdictions found that the prevalence of Internet addiction in men was only slightly higher than that in women (*g* = 0.145) from a global perspective, and that these gender differences in Internet addiction may be partly caused by the gender-related gaps between in economy and in Internet penetration (Su et al., 2019). The fact that our sample came from the capital of China (Beijing), with a high level of economic development and Internet penetration, may be one of the reasons why there were no gender differences in Internet addiction in the present study. Furthermore, there are no gender differences in network age and total Internet usage time in the present study, which could provide supportive evidence for the Internet Availability hypothesis. In addition, the YDQ was used to assess participants’ generalized Internet addiction rather than specific Internet addiction in the current study, which could be another important reason why there were no gender differences in Internet addiction in our sample, because gender may show different effect sizes (magnitude and/or directionality) for specific subtypes of Internet addiction (Su et al., 2019). For example, for online gaming addiction, the rates of men vs. women were 31% vs. 13.1%, while for social networking addiction, the rates of men vs. women were 27.8 and 37.3%, respectively (Tang et al., 2017). Although the gender effect size for generalized Internet addiction was small at 0.15, it was 0.67 for online gaming and 0.10 for social networking sites in a Chinese sample. Meanwhile, there was no significant gender effect size for generalized Internet addiction (*g* = −0.03), but medium gender effect sizes for online gaming (*g* = 0.58) and social networking sites (*g* = −0.42) in a sample from the United States (Tang et al., 2017). In our study, boys spent more time online gaming, and the number of boys who frequently played online games was greater than the number of girls, which could provide some supportive evidence for gender-related differences in subtypes of Internet addiction.

### Direct Relations Between Impulsivity and BIS/BAS With Internet Addiction Across Genders

In the current study, we found that impulsivity and BAS could directly positively predict Internet addiction in boys, whereas impulsivity and BIS could directly positively predict Internet addiction in girls. The results in boys could provide supportive evidence for the dual-system neurobiological model that heightened reward-seeking and deficient impulse control may be a risk factor for adolescent addictive behaviors (Casey et al., 2008; Casey and Jones, 2010). Compared with children and adults, adolescents are characterized by an imbalance between early emerging “bottom–up” systems that show exaggerated reaction to motivational stimuli and later maturing “top–down” cognitive control systems (Casey and Jones, 2010). Both in boys and in girls, impulsivity is positively correlated to poor to–down cognitive control (Casey and Jones, 2010). Meanwhile, with regard to bottom-up motivational stimuli, boys could be more sensitive to reward stimuli (Steinberg et al., 2009), while girls could be more sensitive to punishment stimuli rather than reward stimuli (Pagliaccio et al., 2016). With the rapid development of the Internet technology, 85.3% of adolescents in China have access to the Internet (China Internet Network Information Center [CNNIC], 2016). As a result, adolescents could seek abundantly available rewarding stimuli conveniently on the Internet. Especially for boys with low cognitive control capacity (high impulsivity), these rewarding stimuli (e.g., online gaming positive incentive) continuously reinforce their Internet behaviors, which gradually increase their risk for Internet addiction. Compared with real world, cyberspace could provide a more convenient, anonymous, and safe social interactions environment. Girls with high BIS, who are more sensitive to punishment stimuli (e.g., criticism or scolding from other people), are prone to overuse of the Internet to “escape loneliness” and “belong to a group” instead of face-to-face interactions or offline activities (Park et al., 2013). Furthermore, low cognitive control capacity (high impulsivity) could deteriorate the negative impact of BIS on Internet use in girls. Thus, girls with high impulsivity and high BIS are more prone to Internet addiction than other girls. Taken together, the present study considers the role of gender in the associations among impulsivity, BIS/BAS and Internet addiction according to the dual-system neurobiological model, which could provide further supportive evidence for the key role of impulsivity on adolescent Internet addiction (Crews and Boettiger, 2009; Brand et al., 2014), and could explain the previous inconsistencies in the associations between BIS/BAS and adolescent Internet addiction (Yen et al., 2012; Park et al., 2013).

### Mediating Relations Among Impulsivity, BIS/BAS to Internet Addiction Across Genders

The present study developed a multi-group mediation model to illuminate the different mechanisms underlying the associations of impulsivity and BIS/BAS with Internet addiction between girls and boys. The most important and interesting results showed that different coping styles were an important mechanism through which impulsivity and BIS/BAS were associated with Internet addiction across genders. Specifically, impulsivity and BIS increased the risk of adolescent Internet addiction through enhanced emotion-focused coping in girls. However, impulsivity raised the risk of adolescent Internet addiction through increased emotion-focused coping and decreased problem-focused coping in boys. In addition, problem-focused coping mediated the associations of BAS with Internet addiction in boys. Adolescents have to cope with massive stressors caused by biological, cognitive and social changes that occur across development from childhood to adulthood. The social gender role theory indicates that appraisals of life events might differ across genders (Tamres et al., 2002; Sarrasin et al., 2014). Specifically, compared with men, women are more likely to appraise events as stressful and view stressors as threats rather than challenges. Furthermore, the social gender role theory also indicates that men are socialized as independent, self-reliant and to suppress emotions, while women are socialized as warm, supportive, compassionate, sensitive to the feelings of others, and emotionally expressive with less restrictions (Reevy and Maslach, 2001). Therefore, women are more likely to be accepted by others if they express negative emotions in social interactions than men. Consequently, when the Internet is used as a stress coping resource, girls with high impulsivity and high BIS are more likely to adopt emotion-focused coping (e.g., venting emotions, fantasy, and avoidance) to palliate event-related distress on the Internet, which in turn increases their risk of Internet addiction. These findings are similar to the results of a recent study showing that the combination of impulsivity and neuroticism increases the risk of emotion-focused coping, and thus exacerbates adolescent binge eating behaviors (Keough et al., 2016).

As for boys, when the Internet is used as a stress coping resource, boys with high impulsivity are more likely to use emotion-focused coping (e.g., cyberbullying and avoidance by playing online games) but less likely to engage in problem-focused coping, which is in line with previous studies (Connor-Smith and Flachsbart, 2007; Lee-Winn et al., 2016). Previous studies have also indicated that a person with emotion-focused coping views Internet use in a more dependent manner (Lam et al., 2009; Milani et al., 2009). The virtual world created by the Internet provides them with an opportunity to escape from external stress and difficulties in real life through short-term pleasure and relief. However, problem-focused coping could decrease the risk of Internet addiction (Al-Gamal et al., 2016). Therefore, more emotion-focused coping and less problem-focused coping increases the risk of Internet addiction.

On the other hand, boys with high BAS have high novelty seeking and fun seeking tendencies (Casey and Jones, 2010), which may cause them to use problem-focused coping frequently in stressful environments. When dealing with the external stress and difficulties that occur in real life, the Internet can be seen as a helpful resource (e.g., acquiring information, seeking others help) instead of as an escape for boys with high BAS. This problem-focused coping could decrease their risk for Internet addiction. Thus, high BAS maybe plays an indirect protective role against Internet addiction in boys, which is similar to the results of Hasking’ study showing that reward responsiveness is positively associated with the use of problem-focused coping, which in turn is negatively associated with delinquent behavior in adolescents (Hasking, 2007). Taken together, combined with the direct and indirect relations between BAS and Internet addiction in the present study, these results suggest that BAS is a double-edged sword for Internet addiction in boys, which calls for the need for more studies to understand the beneficial and harmful factors related to BAS in boys.

### Implications for Theory and Practice

From a theoretical perspective, extending previous research, the present study provides empirical support for the dual-system neurobiological model among boys in the context of Internet addiction. Furthermore, our study indicates that the combination of deficient impulse control and heightened BIS rather than BAS increases the risk for Internet addiction in girls. To our knowledge, this is the first study to examine the mediating roles of problem-focused and emotion-focused coping from impulsivity and BIS/BAS to Internet addiction and the moderating role of gender among these associations, which is helpful to improve our understanding of Internet addiction among adolescents. It is especially important in cases where Internet addiction is not viewed as a disorder regardless of category by DSM-V because of insufficient evidence (Grant and Chamberlain, 2016). From a practical perspective, our findings may be helpful for providing evidence-based preventions and interventions to decrease Internet addiction among adolescents. In general, further attention should be given to developing Internet addiction prevention and intervention programs that are tailored to the different needs of girls and boys. First, when screening and choosing a target population for further prevention and intervention programs among adolescents, different combinations of risk factors (e.g., impulsivity and BAS in boys) should be adopted according to gender differences. Second, and even more importantly, our results could offer invaluable knowledge on how to prevent and intervene in Internet addiction among adolescents. Specifically, interventions to decrease impulsivity and BIS could have the potential to decrease Internet addiction in girls, while interventions to reduce impulsivity and BAS may have the potential to decrease Internet addiction in boys. In addition, the finding that coping style mediates the associations from impulsivity and BIS/BAS to Internet addiction across genders provides important implications for practice. To prevent and intervene in Internet addiction in adolescents, training techniques should be exploited to enhance adolescents’ coping style skills because improving specific behaviors may be more efficient than directly altering individual dispositions (Taylor and Stanton, 2007). On the one hand, parents and practitioners should provide a supportive environment, which could play a positive role in adolescents’ coping skills and consequently decrease the risk of Internet addiction in adolescents. On the other hand, coping effectiveness training has previously been confirmed to be an efficient method for improving coping skills (Folkman et al., 1991). Parents and practitioners could adopt methods to help girls decrease their use of emotion-focused coping when managing developmental tasks. In the context of stressors, it would be beneficial to improve the use of problem-focused coping and decrease the use of emotion-focused coping in boys, such as providing boys with the knowledge and practical skills to resolve their problems, seek social support and balance their emotions effectively.

### Limitations and Further Directions

Although this study revealed the gender-specific pathways from impulsivity and BIS/BAS to adolescents’ Internet addiction, several limitations of the current study merit attention. First, the intention of our study is not to “psychopathologize” adolescence, but rather to explore why some adolescents are more vulnerable to Internet addiction than others. However, because Internet addiction is significantly associated with symptoms of depression, social anxiety, attention-deficit/hyperactivity disorder and other mental disorders, which were not investigated in our sample, future studies including these factors could provide stronger evidence for our results with a high capacity to control for these confounding variables. Second, although YDQ and BIS/BAS scales have good reliability and validity in Chinese adolescents (Cao et al., 2007; Li et al., 2014, 2015), the Cronbach’s alpha coefficients for the YDQ and BIS scale were low in the present study, which may be caused by their limited number of items (8 and 7, respectively). Future research should adopt scales with more items to assess Internet addiction and BIS (e.g., the Young’s Internet Addiction Test with 20 items; the Sensitivity to Punishment and Sensitivity to Reward Questionnaire with 48 items) (Torrubia et al., 2001; Widyanto and McMurran, 2004). Third, the present study employed a cross-sectional design. Although this study design can demonstrate strong associations among variables, it cannot provide strong evidence of causal relationships among these variables. Therefore, experimental methods and longitudinal designs could be beneficial in future research to provide more reliable conclusions about the directionality of these effects. Finally, more and more research points toward the type of Internet application (e.g., gaming and social networking site) – not the Internet itself – as being responsible for the development of a problematic usage (van Rooij et al., 2010; Lee et al., 2015). However, the type of usage was not considered, which could pose a limitation in the present study. Furthermore, evidence from previous studies has shown that it is very important to distinguish between generalized Internet addiction and specific Internet addiction and that large gender differences exist in online gaming and social networking site use (Montag et al., 2015; Tang et al., 2017; Lopez-Fernandez, 2018). Further studies are needed to include more Internet usage information, which could provide stronger evidence of whether the results of the current study are appropriate for generalized Internet addiction or specific Internet addiction.

## Data Availability Statement

The datasets generated for this study are available on request to the corresponding author.

## Ethics Statement

The studies involving human participants were reviewed and approved by the Ethics Committee of the Institute of Psychology of the Chinese Academy of Sciences. Written informed consent to participate in this study was provided by the participants’ legal guardian/next of kin.

## Author Contributions

QL and XL designed the study and wrote the protocol. QL and YZ collected the research data. BD conducted the statistical analyses and wrote the manuscript. WD and LW conducted the literature searches and created the figures. All authors approved the final version of the manuscript.

## Conflict of Interest

The authors declare that the research was conducted in the absence of any commercial or financial relationships that could be construed as a potential conflict of interest.
